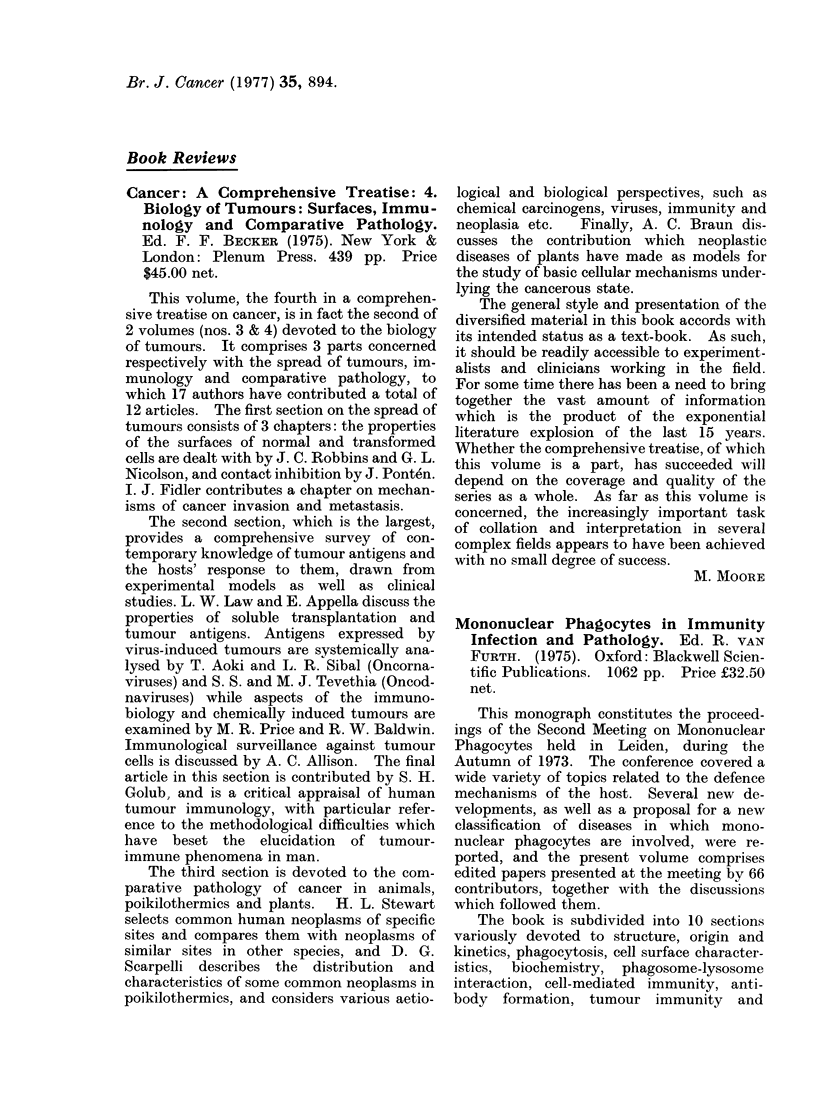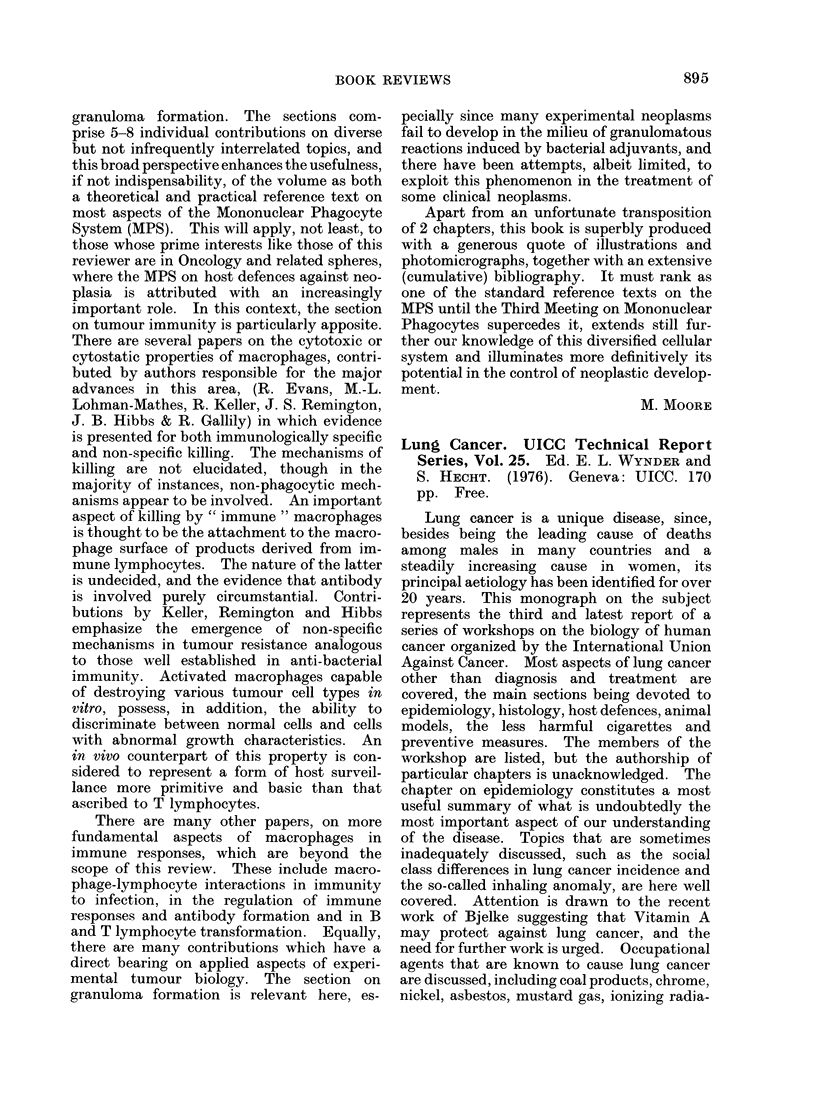# Mononuclear Phagocytes in Immunity Infection and Pathology

**Published:** 1977-06

**Authors:** M. Moore


M. Moore

**Mononuclear Phagocytes in Immunity Infection and Pathology.** Ed. R. van Furth. (1975). Oxford: Blackwell Scientific Publications. 1062 pp. Price £32.50 net.

This monograph constitutes the proceedings of the Second Meeting on Mononuclear Phagocytes held in Leiden, during the Autumn of 1973. The conference covered a wide variety of topics related to the defence mechanisms of the host. Several new developments, as well as a proposal for a new classification of diseases in which mononuclear phagocytes are involved, were reported, and the present volume comprises edited papers presented at the meeting by 66 contributors, together with the discussions which followed them.

The book is subdivided into 10 sections variously devoted to structure, origin and kinetics, phagocytosis, cell surface characteristics, biochemistry, phagosome-lysosome interaction, cell-mediated immunity, antibody formation, tumour immunity and granuloma formation. The sections comprise 5–8 individual contributions on diverse but not infrequently interrelated topics, and this broad perspective enhances the usefulness, if not indispensability, of the volume as both a theoretical and practical reference text on most aspects of the Mononuclear Phagocyte System (MPS). This will apply, not least, to those whose prime interests like those of this reviewer are in Oncology and related spheres, where the MPS on host defences against neoplasia is attributed with an increasingly important role. In this context, the section on tumour immunity is particularly apposite. There are several papers on the cytotoxic or cytostatic properties of macrophages, contributed by authors responsible for the major advances in this area, (R. Evans, M.-L. Lohman-Mathes, R. Keller, J. S. Remington, J. B. Hibbs & R. Gallily) in which evidence is presented for both immunologically specific and non-specific killing. The mechanisms of killing are not elucidated, though in the majority of instances, non-phagocytic mechanisms appear to be involved. An important aspect of killing by " immune " macrophages is thought to be the attachment to the macrophage surface of products derived from immune lymphocytes. The nature of the latter is undecided, and the evidence that antibody is involved purely circumstantial. Contributions by Keller, Remington and Hibbs emphasize the emergence of non-specific mechanisms in tumour resistance analogous to those well established in anti-bacterial immunity. Activated macrophages capable of destroying various tumour cell types *in vitro*, possess, in addition, the ability to discriminate between normal cells and cells with abnormal growth characteristics. An *in vivo* counterpart of this property is considered to represent a form of host surveillance more primitive and basic than that ascribed to T lymphocytes.

There are many other papers, on more fundamental aspects of macrophages in immune responses, which are beyond the scope of this review. These include macrophage-lymphocyte interactions in immunity to infection, in the regulation of immune responses and antibody formation and in B and T lymphocyte transformation. Equally, there are many contributions which have a direct bearing on applied aspects of experimental tumour biology. The section on granuloma formation is relevant here, especially since many experimental neoplasms fail to develop in the milieu of granulomatous reactions induced by bacterial adjuvants, and there have been attempts, albeit limited, to exploit this phenomenon in the treatment of some clinical neoplasms.

Apart from an unfortunate transposition of 2 chapters, this book is superbly produced with a generous quote of illustrations and photomicrographs, together with an extensive (cumulative) bibliography. It must rank as one of the standard reference texts on the MPS until the Third Meeting on Mononuclear Phagocytes supercedes it, extends still further our knowledge of this diversified cellular system and illuminates more definitively its potential in the control of neoplastic development.

M. MOORE